# Self-stigma and its reduction in patients with bipolar affective disorder

**DOI:** 10.1192/j.eurpsy.2024.324

**Published:** 2024-08-27

**Authors:** V. Mitikhin, D. Oshevsky, L. Alieva

**Affiliations:** Mental Health Research Centre, Moscow, Russian Federation

## Abstract

**Introduction:**

The phenomenon of self-stigma in patients with bipolar affective disorder (BD) has been studied much less than in other mental disorders (Favre, Richard-Lepourie, 2023). However, self-stigma has equally negative psychosocial consequences for them (Shargh et al, 2015). Therefore, identifying the clinical and psychological characteristics of self-stigma in BD patients, especially in the initial stages of the disease, and developing on this basis new directions for their psychosocial rehabilitation to reduce self-stigma is relevant.

**Objectives:**

To identify clinical and psychological characteristics of self-stigma in BD patients, to identify targets for psychosocial rehabilitation.

**Methods:**

«Questionnaire for assessing the phenomenon of self-stigmatization of mentally ill people» (Mikhailova et al., 2005), «Insight Scale for Psychosis» - ISP (Birchwood et al., 1994). We examined 17 patients (12 women and 5 men) with a diagnosis of bipolar affective disorder (F31.xxx according to ICD-10). The average age of the patients was 25.52±4.55 years. The duration of the disorder is 0.5-3 years.

**Results:**

It was shown, that patients with BD had a high level of self-stigma. Indicator «General level of self-stigma» was 1.22±0.73 points, that higher its average values. The main component in the structure of self-stigmatization was an overestimation of possible limitations of one’s own internal activity and self-realization (1.96 ± 0.87 and 1.62 ± 0.82 points, respectively) associated with the disease. Idealization of one’s pre-illness qualities and achievements (1.62±0.82 points) and the formation of misconceptions about the loss of previous opportunities will able to lead to negative personal changes and limit the activity of patients. Correlation analysis revealed significant (p≤0.01) correlations between the «Patient’s ability to recognize painful phenomena as symptoms of mental illness» scale of the ISP scale and individual parameters of the questionnaire for assessing self-stigma: «Imagination of one’s own failure due to illness» - r = 0, 52; «Fear of becoming insolvent due to illness» - r=0.54; «Idealization of the «healthy self», r=0.51. Thus, in BD patients, self-stigma is associated with low awareness of the disease and misconceptions about it and about themselves.

**Conclusions:**

Psychoeducation programs, aimed at formation an adequate perception of mental disorder, the ability to recognize its symptoms, and destigmatization trainings to increase the social activity are needed for BD patients. Such trainings were developed during the research and are currently being tested.

**Disclosure of Interest:**

None Declared

